# The geography of sentiment towards the Women’s March of 2017

**DOI:** 10.1371/journal.pone.0233994

**Published:** 2020-06-04

**Authors:** Diane H. Felmlee, Justine I. Blanford, Stephen A. Matthews, Alan M. MacEachren

**Affiliations:** 1 Department of Sociology and Criminology, Pennsylvania State University, State College, Pennsylvania, United States of America; 2 Population Research Institute, Pennsylvania State University, State College, Pennsylvania, United States of America; 3 Department of Geography, Pennsylvania State University, State College, Pennsylvania, United States of America; 4 Dutton e-Education Institution, Pennsylvania State University, State College, Pennsylvania, United States of America; 5 Department of Anthropology, Pennsylvania State University, State College, Pennsylvania, United States of America; University of North Carolina at Charlotte, UNITED STATES

## Abstract

The Women’s March of 2017 generated unprecedented levels of participation in the largest, single day, protest in history to date. The marchers protested the election of President Donald Trump and rallied in support of several civil issues such as women’s rights. “Sister marches” evolved in at least 680 locations across the United States. Both positive and negative reactions to the March found their way into social media, with criticism stemming from certain, conservative, political sources and other groups. In this study, we investigate the extent to which this notable, historic event influenced sentiment on Twitter, and the degree to which responses differed by geographic area within the continental U.S. Tweets about the event rose to an impressive peak of over 12% of all geo-located tweets by mid-day of the March, Jan. 21. Messages included in tweets associated with the March tended to be positive in sentiment, on average, with a mean of 0.34 and a median of 0.07 on a scale of -4 to +4. In fact, tweets associated with the March were more positive than all other geo-located tweets during the day of the March. Exceptions to this pattern of positive sentiment occurred only in seven metropolitan areas, most of which involved very small numbers of tweets. Little evidence surfaced of extensive patterns of negative, aggressive messages towards the event in this set of tweets. Given the widespread nature of online harassment and sexist tweets, more generally, the results are notable. In sum, online reactions to the March on this social media platform suggest that this modern arm of the Women’s Movement received considerable, virtual support across the country.

## Introduction

On Jan. 21, 2017, millions took to the street in an historic event, the Women’s March of 2017, which at the time represented the largest, single-day protest in American history. Estimates suggest that the March involved over 4.1 million people in the United States, comprising about 1.3 percent of the U.S. population [1]. Demonstrators protested the inauguration of President Trump and rallied around issues such as rights for women, reproduction, and immigration and civil rights [2,3]. One of the goals of the event was to generate a supportive atmosphere for women and their causes, and tremendous enthusiasm was reported, with the theme “Hear our voice” underscored by countless speakers and demonstrators. Not unlike other large, protest movements, however, the event also invoked controversy. Criticism and harassment arose from the political right and certain activist factions; this activity was reflected in social media.

Little systematic research has examined public reactions to this historic event, and the question remains whether a peaceful, extensive March such as this one can affect those reactions. With the advent of social media, we can begin to address such a question in a manner not available in earlier periods. The purpose of this exploratory study, therefore, is to investigate the extent to which this single protest influenced the sentiment of the public, either positively or negatively, as reflected in the social media forum of Twitter. Findings are relevant for other rallies and social movements. If such a sizeable event can generate largely supportive commentary, rather than harassment and resentment, for example, alternative organizations might be able to learn from its success.

The Women’s March of 2017 also had a wide-spread, geographic stamp within the nation and abroad. In an act of transnational solidarity, women’s marches sprung up world-wide, with at least 273 events in eighty-two countries, with worldwide participation estimated at over seven million. Within the U.S., estimates reported at least 680 “sister marches” in a range of locales, with large groups taking to the streets in major metropolitan areas, but mobilization also occurring in less densely populated states, such as Wyoming and Alaska [1]. While it is logical to hypothesize that sentiment about the March differed geographically, research to explore that variation is lacking. Support (or resistance) towards the event, for example, could be concentrated largely within the main locale for the March, the Washington DC area, rather than extend far beyond those boundaries. Alternatively, sentiment could align along political, geographical boundaries, with positive responses relegated to liberal areas. A key question we examine in our research, therefore, is the following: To what extent do emotional responses to the March expressed on Twitter vary by geographical locale?

In this study we investigate a sample of approximately 2.45 million geo-located tweets gathered prior to, during, and following the day of the Women’s March. We employ a sentiment classifier to analyze the degree of positivity/negativity of tweets concerning the March (and sister marches) and about females, more generally. The tasks of the research are as follows:

1. Investigate variation in sentiment by geographical location.
2. Examine changes over time in the degree of positivity of sentiment in tweets, and test whether the March, itself, influenced sentiment towards the March
3. Identify the most positive and negative geographic areas in Twitter responses to the March.

### Women’s March of 2017

The 2017 Women’s March on Washington began with a post on Facebook in which a retired attorney in Hawaii, Teresa Shook, invited friends to march on Washington as a sign of protest following the November election; by the next morning, 10,000 women had registered support for March [2]. Additional organizers throughout the country joined the call-to-action with resounding success. As noted earlier, the March involved over four million women and their allies and resulted in multiple sister marches reaching across the U.S. and beyond. Over 500,000 marchers swelled the streets of Washington DC alone, with thousands participating in other large urban centers.

The goals of the March evolved out of a set of values and principles that included the basic tenet that women’s rights are human rights. A set of eight “Unity Principles” aimed to be inclusive of a range of intersecting race and cultural identities and sought to affirm shared humanity and a commitment to resistance. The eight Unity Principles included the following: ending violence; reproductive rights; LBGTQIA+ (lesbian, bisexual, gay, transsexual, queer, intersex, and asexual) rights; worker’s rights; civil rights; disability rights; immigrant rights; and environmental justice [2].

The diverse principles espoused by the March organizers demonstrated what has been termed boundary-spanning characteristics of a social movement [4]. On the day of the March, the Women’s March website included more than 400 organizational partners [5], highlighting the role of joint resource mobilization [6] in spurring the event. As found in previous women’s movement events [7], moreover, feminist activist networks within the U.S. federal bureaucracy played a part in generating participation, with several organizers representing varied government organizations. The protest brought together participants representing an assortment of diverse goals that were not limited to women’s rights, as evident in survey responses [5], and in messages on protest-signs in Washington DC [8]. Furthermore, the event addressed the oft-asked question: What has happened to the Women’s Movement? [9]. As suggested by several scholars (e.g., [10]), the Women’s Movement did not die out as some feared; instead it retrenched, extended into numerous, new arenas, and then re-emerged on a large and multi-focused scale at this particular moment in time.

Several conservative commentators launched criticisms at the March and its goals. On her national TV show, Tomi Lahren, for example, called the marchers “a bunch of sore losers” who were throwing a “tantrum,” and charged that there was an effort to silence conservative women. Within the movement, disputes arose. Complaints cropped up concerning the name of the March (originally labelled the “Million Women’s March”), the goals of the March (described as too unrealistic and too narrow [11]; or too broad), issues of unequal representation among women of color and other groups, the role of men, and the status of a permit and location for the March. Despite these controversies, however, the 2017 Women’s March was widely heralded in the press as an enormous and successful feat, with huge crowds endorsing the protest (e.g., [12]). Given the importance of social media to both reflect and influence society today, however, it is important to look beyond reports by traditional media to understand reactions to the Marches within social media. Since most Twitter posts are public, and the current President directly utilizes Twitter to announce decisions and attempt to influence society, a detailed analysis of responses to the Marches in Twitter is a key starting point for understanding emotional response within social media.

### Twitter and public sentiment

Twitter represents one of the most frequently used types of social media, with an average of 500 million tweets sent per day, and 330 million active users per month [13]. Of particular relevance to our study, Twitter messages can be used to capture aspects of public sentiment. For example, O’Connor, Balasubramanyan, Routledge, and Smith [14] find that consumer confidence and political opinion tend to correlate highly with sentiment word frequencies taken from Twitter messages. The content of tweets also reflects public mood as people react in real time to specific social, political, cultural and economic events [15].

Sentiment on Twitter captures not only inoffensive public reactions, but also hostility, aggression, and abuse. Approximately 15,000 intentionally harmful messages transpire on Twitter daily, with close to 100,000 aggressive, bullying comments occurring on a weekly basis [16]. In addition, close to 10,000 racial slurs are posted on Twitter per day [17], with 30 percent of them employed in a clearly pejorative manner. These negative messages are disturbing not only in content, but because they have the potential to spread widely in an online network of retweets, likes, and mentions (e.g., [18]). Furthermore, women constitute a common target of harassment (e.g., [19]) in tweets that reinforce derogatory, female stereotypes [20,21] with the use of several frequent, sexist curse words (e.g., “b!tch,” “c!nt,” “sl!t”) used on Twitter [22].

The purpose of this study is to examine the degree to which support for the rally succeeded in buoying public sentiment in a positive direction. However, we also will consider whether the political event spurred a backlash of negativity on Twitter, which combined with sexist forms of harassment, could have dominated communication during the period of the March. In addition, we investigate patterns in hashtag labels associated with the March and examine geographic variation in the sentiment of tweets relevant to those hashtags.

### Geography of sentiment

Geographic variation in sentiment expressed in Twitter (and similar public social media) has attracted the attention of multiple researchers from both the data science [23–26] and the social and behavioral science communities (e.g., [27–30]). Sentiment has been interpreted in a range of ways (as a measure of opinion, happiness, well-being) and has been measured using a wide range of methods; an overview of the interpretations and metrics is provided below. Thus, comparison across studies is difficult (and sometimes not possible). The volume of research on geographic aspects of sentiment would require a full review paper to synthesize; for example, the following pair of query phrases submitted together in Google Scholar produced 451 papers: "geographic distribution," "sentiment analysis". Here, we focus on two key topics within this research domain that are directly relevant to the research we report: (a) how sentiment is measured and validated (and the range in accuracy reported when validation is carried out), and (b) the contexts, objectives, and limitations associated with and insights derived by measuring geographic variation in sentiment.

### Sentiment measurement and validation

Sentiment measurement methods can be classified in multiple ways. Beigi, et al. [23] make a high-level distinction between language-processing-based and application oriented methods. Zou, et al [31] present essentially the same distinction with the second category called “classification-based”. Within the first category, Beigi and colleagues subdivide further into lexicon-based methods and linguistic analysis. They go on to recognize two subcategories of lexicon-based methods: (a) dictionary-based in which a set of words is determined initially and a bootstrapping approach is used to extend the list and (b) corpus-based, in which the lexicon is generated by learning the dataset. Complementing the lexicon-based approach, linguistic methods make use of grammatical structures to support the classification. In contrast to the language-processing based methods, the application-oriented (or classification-based) methods apply machine learning strategies to learn the distinction between binary or multi-class sentiment for text within some application domain (e.g., product reviews, reactions to natural disaster events, etc.). The lexicon-based methods are, necessarily language dependent. In contrast, classification based methods have been developed that are language independent (e.g., Davies and Ghahramani report on a Bayesian sentiment mining method designed to assess happiness from tweets, independently of the language the tweet is in [32]).

In research that considers geographic variation in sentiment (or its components), few studies use classification-based approaches to sentiment analysis; most use lexicon-based methods that leverage a dictionary. For Twitter, specifically, VADER is a popular lexicon-based method that has scored high on comparative assessments against other methods [33,34]. However, we found fewer instances of its application to sentiment analysis focused on geographic variation. One exception is [31] who apply VADER (using its default lexicon with no reported extension) in the context of analyzing sentiment related to stages in a disaster event.

Perhaps not surprisingly, since few studies attempt to refine the lexicon-based sentiment analysis methods by extending their dictionaries (or through any other means), we find a relatively small number of studies that report a validation process or results. Most take for granted that the methods produce useful results (relying only on the original validation reported by VADER’s authors); this is in spite of evidence that is reported showing accuracy no higher than the mid-80% range. When validation is reported, it has often lacked details. In one of the few studies to report a rigorous validation, Nguyen, et al. [35], use online crowdsourcing to manually label 500 tweets”, with 15 raters each, as “happy”, “sad” or “neutral. As with other studies that include validation, they do not report inter-rater reliability, nor do they say whether they accepted majority vote or used some other method to decide on the rating of any tweet with substantial disagreement. They report 73% accuracy of their “sentiment” analysis in comparison to this human-generated data; their method was specifically targeted to categorizing tweets into happy, sad, and neutral using the Language Assessment by Mechanical Turk (LabMT) word list plus a set of emoticons and punctuation that signifies emotion.

Beyond the individual studies that report validation of sentiment analysis methods in the context of research that addresses geographic aspects of sentiment, Beigi, et al. [23] provide a broad overview of sentiment analysis as it has been applied to social media. As part of that review, they include a table that summarizes 14 recent studies, providing information on the dataset analyzed, the approach to measuring sentiment (which includes lexical and machine learning methods), and results of evaluation (with some reporting F-score, that ranges from 58% to 86%, accuracy, that ranges from 70% to 84%, plus one that used RMSE, with 0.49 reported).

### Contexts, objectives, and limitations for social media geo-sentiment analysis and insights

Geolocated tweets have been recognized as a potential source of information about behavior and attitudes of peoples in a wide range of problem contexts. Some of the earliest work focused on the potential to use geolocated data from twitter to support crisis situation awareness and response (e.g., [36–38]). Most of this work relies on data from explicitly geolocated tweets in which the Twitter user turns “location” on (about 1–2% of all tweets, [39]). Additionally, some of this research has geolocated places mentioned in the tweet text (e.g., [36]) and locations from the Twitter user’s profile (e.g., [40]).

Several studies with a focus on crisis events have explored the potential to use sentiment analysis as input to various stages in crisis management [41,42]. In one of the more comprehensive studies of geolocated sentiment related to crisis events, Zou, et al. [31] explore the potential to use Twitter data to support not only disaster damage assessment but also how integrating twitter sentiment analysis with environmental and socioeconomic variables can generate insights and support strategy development toward creation of more resilient communities. They present a series of county-level maps showing that sentiment is most negative during hurricane response (for Hurricane Sandy), particularly in the locations with the most impact from the storm. The most positive sentiment occurred during recovery, with a small set of negative locations remaining negative and a few that switched from positive to negative.

In related work, Wendland, et al. [43], apply the “Appraisal System” developed by Martin and White [44] to carry out a sophisticated form of sentiment analysis for tweets related to a hostage event in Australia, the Sydney Siege of 2014. This system divides “attitude” into subcategories of “affect”, “judgement”, and “appreciation”, with each of these subdivided into subtler distinctions (e.g., affect is broken down into un/happiness, in/security, and dis/satisfaction). They apply the system to exploring these various components of sentiment by Twitter users both during and after the event. Analysis considers how time of day of event components as it relates to time of day at the Twitter user’s location influences both frequency and content of tweets. They examine the frequency of these different components of sentiment overall, in relation to the victims, in relation to Islam (given the assumption at the time that this was an Islamic-related terrorist attack), and in relation to the hashtag of #ILLRIDEWITHYOU (associated with an effort toward community cohesion and opposition to fear concerning Muslims). Results indicated a more positive sentiment than anticipated, in part due to may tweets about hopes and prayers about a positive outcome being rated as positive. Unexpected results also included a relatively low incidence of anti-Muslim sentiment and the development of hashtags linked to positive social action intended to enhance community cohesion in the future.

As noted above, a frequently studied topic using sentiment analysis methods applied to tweets is happiness. Here, we focus on some of the research questions addressed and insights derived. Most of the studies of happiness using tweets as a data source have related a derived happiness index/rating to socioeconomic or other characteristics of places to which tweets are aggregated. The best know study is probably the one by Mitchell, et al., [27]. They introduce a word-shift graph technique that is used to explore differences in frequency of words associated with happiness or sadness for places compared to their overall frequency. Based on their analysis, they contend that Hawaii is the happiest state (numerically), due to inclusion of words like “beach” and food-related terms. But, it seems like a stretch to count mentions of things that tourists are likely to talk about as an indication of happiness of the state. The authors report results of correlation analysis between happiness and cluster-grouped demographic attributes for 373 cities. They find that happiness correlates positively with wealth and negatively with obesity. They also present an interesting cross-correlation analysis for word frequency distributions for 40 cities, with cities such as Baton Rouge, New Orleans, and Memphis (not surprisingly) being among the most similar and those cities exhibiting high contrast with Milwaukee, Orlando, and Austin.

A recent paper by Jensen [45] provides a detailed critical analysis of the Mitchell happiness study. While focused on that one study, the issues raised about representativeness of social media data and other concerns with its use are relevant for all research that attempts to leverage these data in support of social science research about populations. Issues discussed include the relatively low proportion of the population that uses twitter, that the users tend to be older than users of some other social media and that twitter users who chose to turn location on may not be representative of Twitter users overall. A particularly important point in relation to research that attempts to answer questions about behavior of people is that on-line and off-line social behavior are likely to differ. This, of course, is not an issue when the focus of research is specifically on-line behavior, as it is in research reported here.

In addition to the research synthesized above, there has been a substantial body of work on sentiment visualization, some of which has targeted geographic variation in sentiment. Interested readers can consult Kucher, et al. [25] for a wide-ranging review of this visualization research that contains discussion of 14 papers that specifically include a map component as part of the visualization approach.

## Methods

In our study we examined the content of messages about the 2017 March and its sister marches, including where these occurred in the United States (excluding Alaska and Hawaii). Geo-located tweets were collected for the entire continental U.S. using the Twitter Streaming Application Programming Interface (API) [46]. A filter was set to collect geocoded tweets within 180° West Longitude to 30° West Longitude and 6° North Latitude to 90° North Latitude to cover all of North America. Tweets were collected for the 20^th^, 21^st^ and 22^nd^ January 2017 and saved to a text file in JSON format using a node.js application [47] (N = ~2.45 million (N = 2,448,428)). Thirteen percent of messages had latitude and longitude for a point location while the remainder, although geo-located, represented a geographic bounding box. For locations with a bounding box, the centroid of the box was calculated and used to represent the geographic location for that tweet. This research was conducted with the formal approval of the Pennsylvania State University Institutional Review Board, and the Review Board waived the requirement for informed consent. Our methods of data collection complied with the terms and conditions specified by the website.

Messages about the March were identified using terms specific to the event (i.e., *women*, *march*, *women(s)march*, *woman(s)march*). We searched for these terms, and for combinations of the terms, to identify tweets related to the March. Tweets were collected for the dates before and after the March, and retweets were not included. Since we were interested in motivations and perceptions about the March, we examined (i) hashtags to understand why people were marching and (ii) sentiment to better understand the context of each message associated with the March; that is, whether the message being conveyed was positive or negative in nature.

### Hashtags

Hashtags were extracted from each message and separated into three groups: womensmarch, geography and whymarch. *Womensmarch* includes hashtags that contained (*women(s)march*, *woman(s)march*, *women and march*). *Geography* of the marches were obtained by extracting place from hashtags that contained (*women(s)march*, *woman(s)march*, *march*), since many locations were included at either the start or end of the hashtag (e.g. *womensmarchboston*, *bostonwomesnmarch*) or as a place name included as a hashtag in tweets associated with the march (e.g. *#boston*). *Whymarch* hashtags were all remaining hashtags that did not contain ‘womensmarch’ hashtags or geography but were also included in messages about the March (e.g. #*notmypresident*). The total number of times a hashtag was mentioned was calculated for each of the aforementioned categories. Results were visualized using wordcloud in R (V 3.4.4).

### Sentiment

To assess the sentiment associated with the March, the content of each tweet was analyzed using a sentiment classifier that assigned a sentiment score to the message. The classifier we use here incorporates a lexicon approach that leverages dictionaries of words. Since the optimal choice of lexicon depends on the particular data, we chose a classifier that was developed for use with social media data and modified it. Our approach builds on the lexicon created for the VADER (Valence Aware Dictionary and sEntiment Reasoner) classifier, as has been done elsewhere [21]. VADER is “a lexicon and rule-based sentiment analysis tool that is *specifically attuned to sentiments expressed in social media* (fully open-sourced under the [MIT License]” [48]. For this project, we updated VADER’s lexicon to include terms targeted towards females that we found in our manual examination of a random sample of 400 tweets downloaded from the day of the Women’s March. Next, we translated Vader’s score -1 (most negative) to 1 (most positive) score into a -4 (most negative) to 4 (most positive) scale. We used this scale to reproduce the original lexicon scoring used by VADER and to standardize the score across our final classifier (see below). Note that a score of “0” represents neutral or a mix of positive and negative sentiment in a tweet.

To further increase the overall accuracy of the sentiment score, we compared the performance of VADER’s lexicon with several other commonly used lexicons. In the end, we used a combination of scores from the top three lexicons in ensemble, which included VADER and two others, AFINN [49] and Bing [50]. We compared the sentiment scores gathered from three human coders on a test set of an additional 600 tweets with the scores obtained by our final classifier using a 10-fold cross-validation. Our sentiment classifier performed well, with overall F1 scores of 0.746 (micro) and 0.697 (macro). The F1 score represents a weighted harmonic mean of the precision and recall of the instrument, where the maximum value is 1 and the lowest is 0. These F1 scores represented an improvement over scores from VADER’s default classifier alone or from other combinations of the other common classifiers. Sentiment was assessed for (i) all tweets, (ii) tweets associated with the Women’s March on the day of the march, (iii) tweets including hashtags associated with the Women’s March and (iv) tweets including hashtags associated with whymarch.

### Women’s March by geographic location

The reactions to the March in Twitter were also assessed geographically. All tweets that were identified to be about the March were used to create a density map to show twitter activity in locations where women’s marches took place. Place mentions associated with the March were extracted from each hashtag and the median latitude and longitude for all locations associated with the same place name were calculated and used to represent that March. Since place names could be misspelled or abbreviated (e.g. *Washington*, *DC*, *WashingtonDC*, *Washintin*), each place name was analyzed and often assigned a common name (e.g. WashingtonDC for the example highlighted) to include several alternate spellings. For each place name, the total number of mentions was calculated and used to highlight the number of times that place was mentioned during the March and mapped.

Secondly, we assessed the sentiment of each tweet by metropolitan area (MA) (areas with a population greater than 50,000 people and/or containing a state capital) to determine whether some locations were associated with more negative tweets than others. To do so, the mean and median sentiment for each MA were calculated. Boxplots capturing the variability of sentiment for each MA were calculated and used to further assess sentiment by location. Boundaries for MA’s were obtained from the United States Census Bureau [51]. Boxplots capturing the variability of sentiment associated with hashtags linked to whymarch also were determined. Since the number of hashtags associated with this class was large, only hashtags with more than fifty mentions were analyzed. All maps were generated using ArcGIS 10.4 and statistical analyses conducted using R (v 1.3.1).

## Results

### Tweets

Between January 20 and January 22, 3.1% of all tweets analyzed in this study contained a term about the Women’s March. On the day of the March (January 21), 40,174 unique users sent 64,653 geotagged messages about the Women’s March, which comprised 2.6% of all tweets.

### Geographic distribution

**Fig 1:**
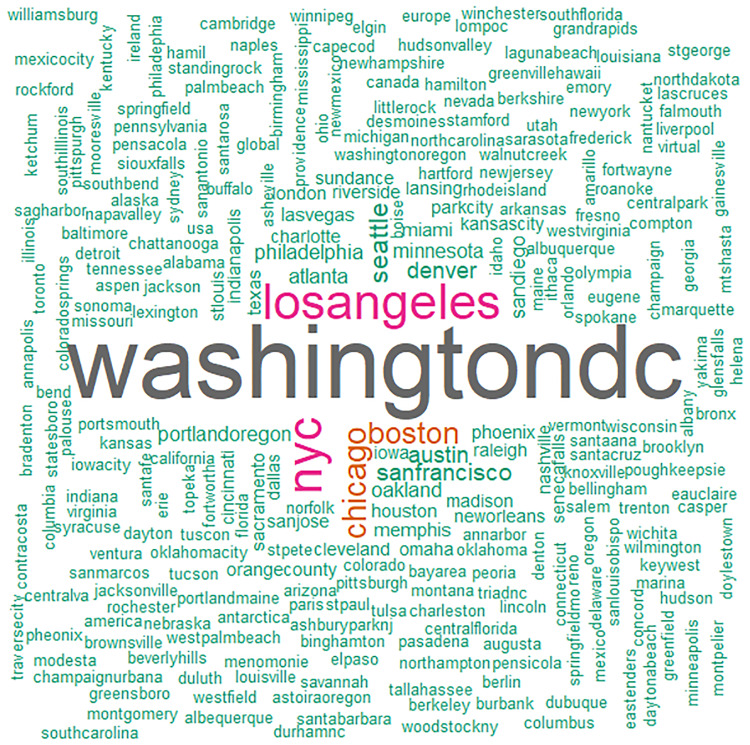
Hashtags with place names mentioned in messages associated with the Women’s March and sister marches.

The largest fifteen marches included major cities from metropolitan areas across the U.S., including the East Coast (e.g., Washington D.C., New York City, Boston, Philadelphia), West Coast, (e.g., Los Angeles, Seattle, San Francisco/Oakland, Portland, San Diego), the Midwest (e.g., Chicago, Madison), the North (e.g., Minneapolis/St. Paul), the Mountain West (e.g., Denver) and the South (e.g., Atlanta, Austin) (see Table 1). Our sample of tweets reflects this wide geographic distribution. In the geo-located tweets relevant to the March, between the January 20 and January 22, 60% of the messages contained hashtags with place names. The hashtags contained over 274 geographic names for locations, as depicted in the wordcloud in Fig 1. Note that the wording of several cities appears in various spellings, misspellings, and acronyms, such as “nyc” and “newyork,” which means that certain cities were more prominent in tweets than it appears. Yet, as evident in the largest names in the wordcloud, the top locations mentioned in tweets, and/or the locations tweeted from, tend to coincide with the sites of the largest marches, such as the top three sites, Washington DC (“washington”), Los Angeles (“la”), and New York City (“nyc”).

**Table 1:** Summary of the largest top fifteen women’s march.

March location	Estimated number of people at march*
Washington D.C.	1,000,000
Los Angeles	750,000
New York City	400,000
Chicago	250,000
Denver	100,000–200,000
Boston	150,000–175,000
Seattle	175,000
San Francisco and Oakland	100,000–150,000
St. Paul, Minneapolis	90,000–100,000
Portland, Oregon	100,000
Madison	75,000–100,000
Atlanta	60,000
San Diego	40,000–50,000
Philadelphia	50,000
Austin	40,000–50,000

*Estimates obtained from Wikipedia.

**Fig 2:**
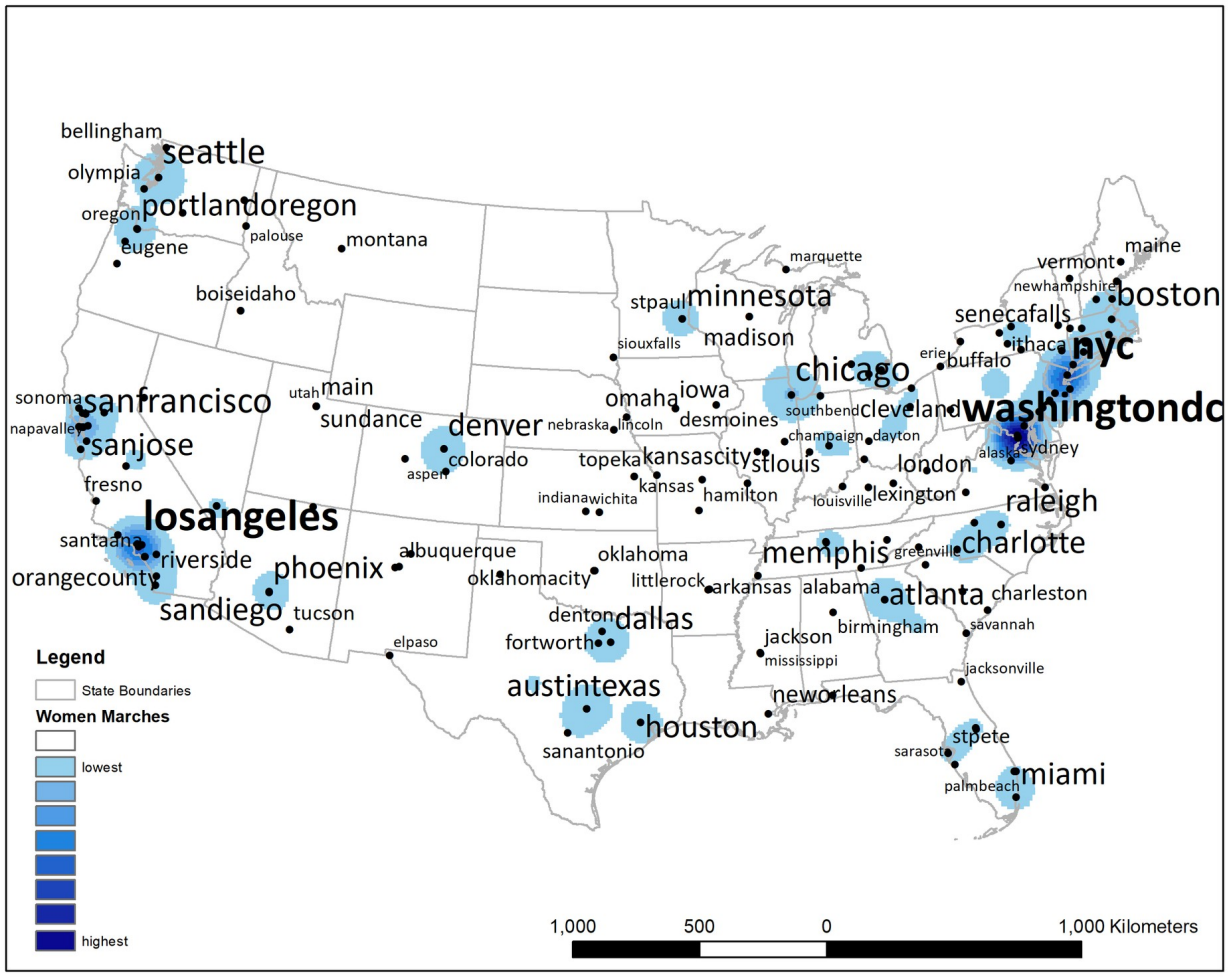
Density of messages sent about the Women’s March and sister marches. The size of text captures number of times a specific place was mentioned. The location of the place name is based on the median location of all mentions of that place name. That is, the median location is not necessarily the actual location of the sister march, and some marches occurred outside of the United States.

As further evidence of the geographic spread of the Women’s March of 2017, the main cities associated with each sister march are depicted in Fig 2. Furthermore, in Fig 2 we see the wide geographic variation in the frequency of tweets, with the density of all tweets relevant to the march superimposed on each city. The darkest areas, representing the highest tweet density, again represent sites of the most highly populated women’s marches, such as Washington DC, Los Angeles, and New York City. In general, the density of tweets tends to reflect the level of turnout for a sister march, although there are exceptions (e.g., Madison, WI was the site of the 11^th^ largest sister March in the country). Such exceptions highlight that population size, as well as the size of the local March, both contribute to tweet density.

### Hashtags

**Fig 3:**
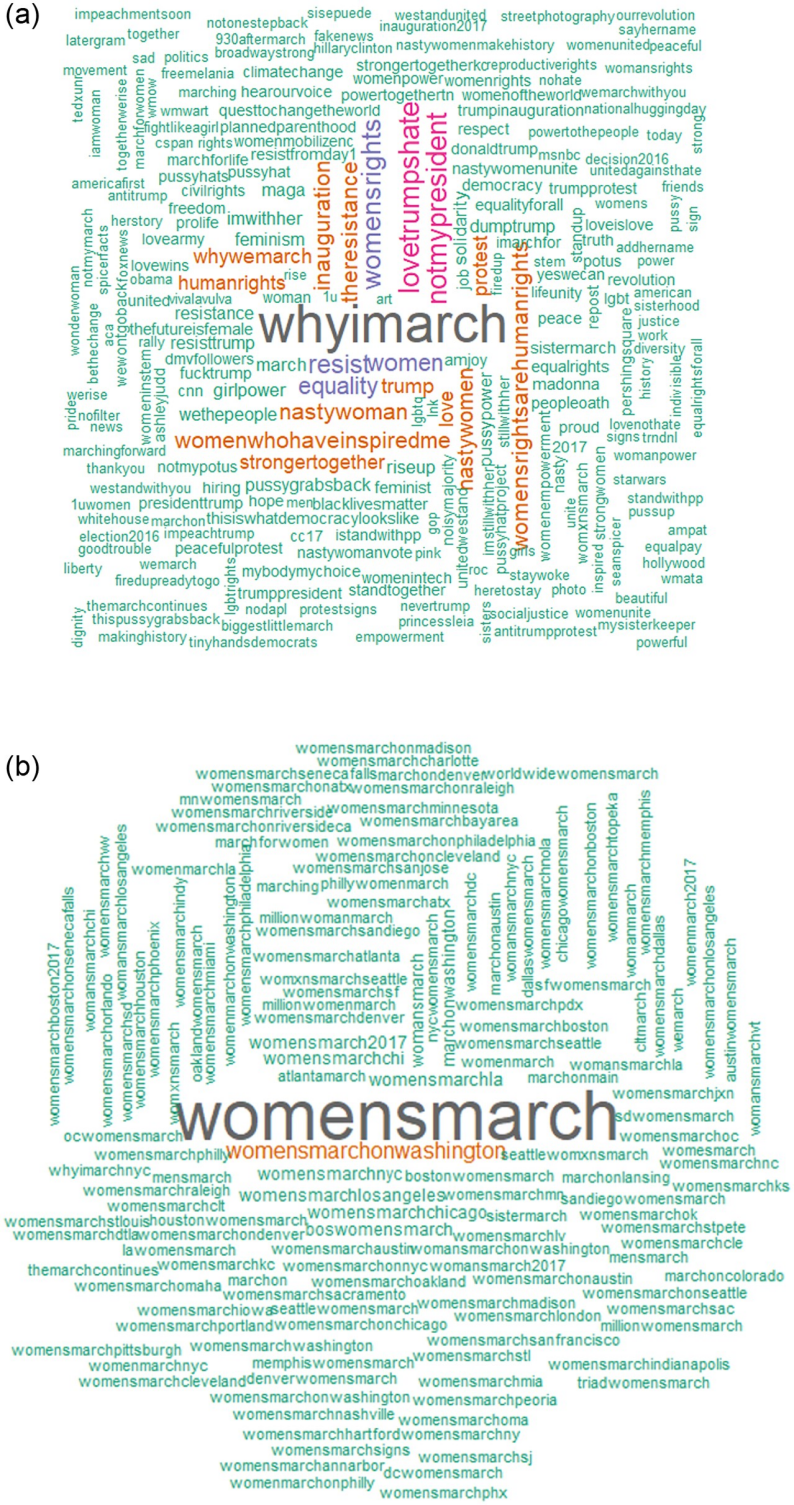
(A) Hashtags mentioned fifteen or more times associated with the marches and (B) place names mentioned in messages associated with the marches.

Over 700 variations of the hashtag *‘womenmarch*, *womanmarch*, *womensmarch or womansmarch*’ (S1 Table, Fig 3A) were used to represent the event. Most of these hashtags identified various locations associated with the March (e.g., womensmarchsacramento, womensmarchpeoria, mnwomensmarch). In addition, over 5,000 hashtags captured reasons for marching (Fig 3B shows all hashtags with a frequency > 15). Some of the most common hashtags in this genre included “notmypresident,” lovetrumpshate,” and “womensrights.”

### Tweet sentiment regarding the March

**Fig 4:**
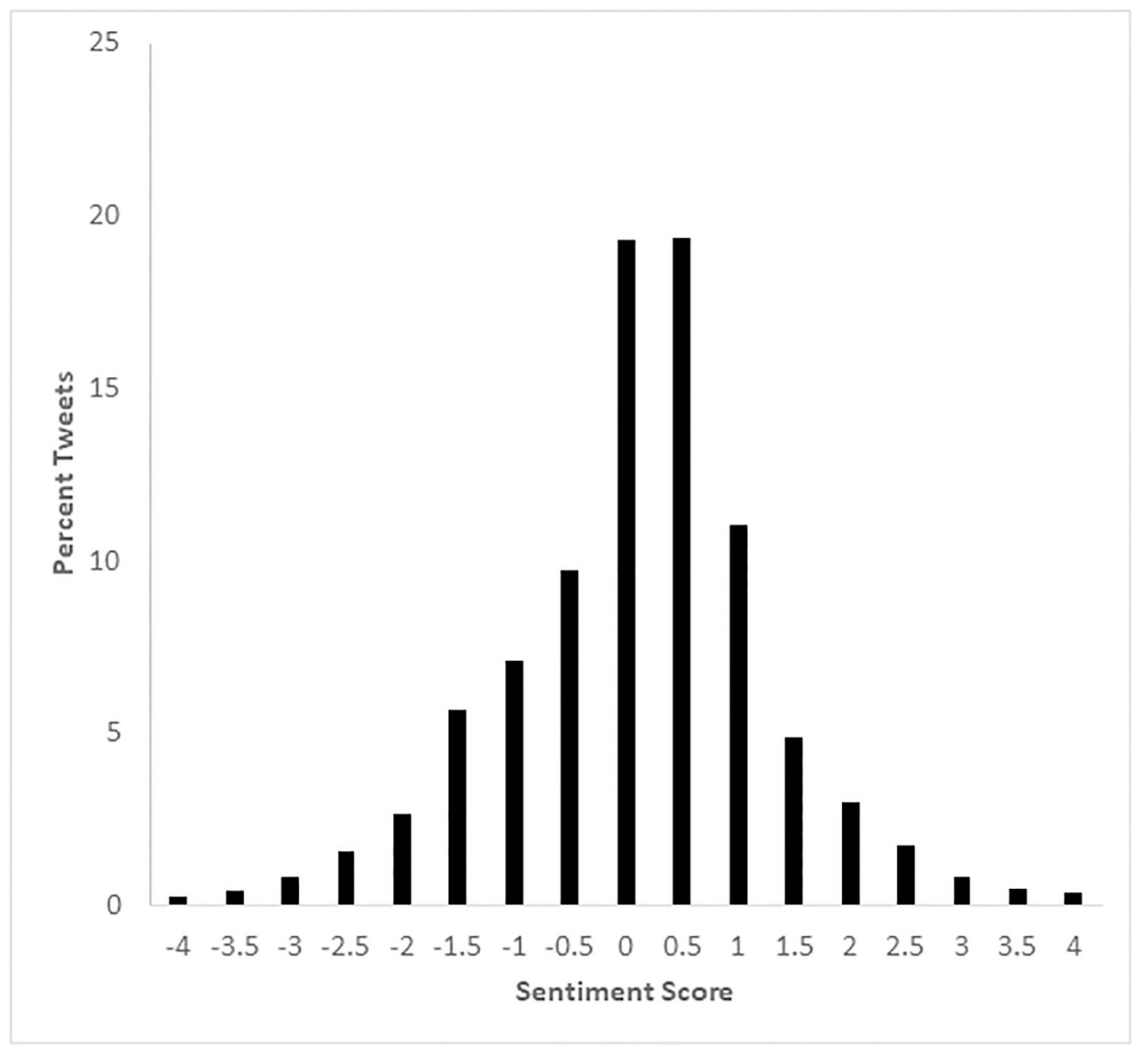
The overall distribution of sentiment for all tweets collected for a 48-hour period on January 20–22, 2017, including the day of the Women’s March (January 21, 2017).

**Fig 5:**
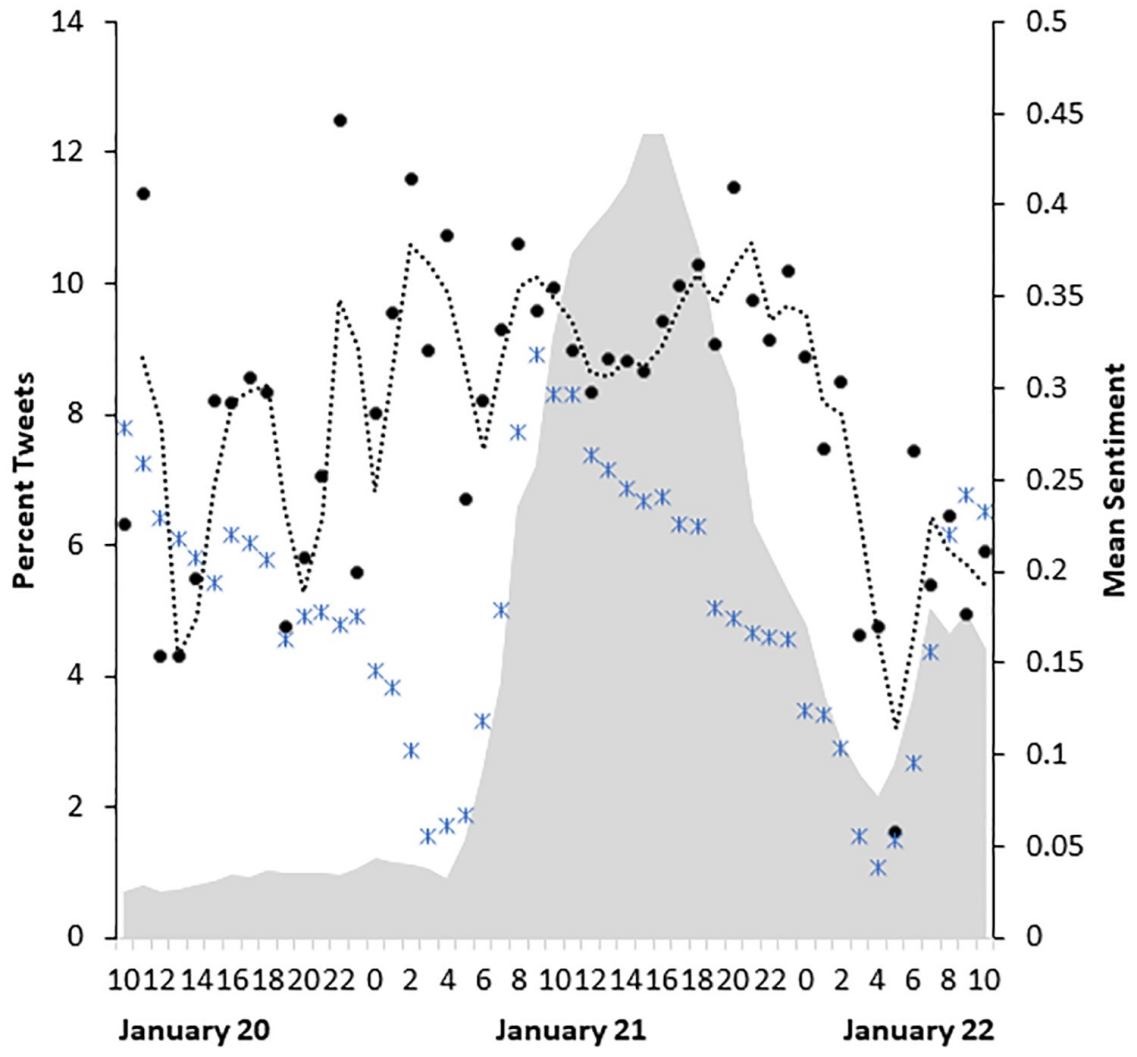
The average hourly sentiment for all tweets (blue crosses) and those associated with the Women’s March (black dots), and percent of tweets about the March (grey shading) collected for a 48-hour period on January 20–22, 2017, including the day of the Women’s March (January 21, 2017).

Selected examples of sentiment scores (-4 to +4) assigned to tweets relating to the Women’s March are provided in Table 2. The distribution of sentiment in all tweets during the period of the study (January 20 to January 22) was approximately normal, with a mean of 0.19 and a standard deviation of 1.03 (Fig 4). The overall sentiment about the March tended to be positive, with a mean of 0.29 (and a standard deviation of 1.09) rising to 0.34 (standard deviation = 1.06) on the day of the March. Messages about the March were most prevalent on the day of the March, January 21 starting at 5am, peaking at 6pm (grey area in Fig 5), and reaching a low at 5am on January 22. Furthermore, sentiment in tweets regarding the March remained more positive than all other geo-located messages sent during the same time on the day of the March, as depicted in Fig 5. Note that the mean hourly sentiment for tweets about the March (black dots) is higher than the mean hourly sentiment for all geolocated tweets (blue “x”) sent during that same time period (see Fig 5). The black dotted line in the figure indicates the smoothed, moving average of tweet sentiment for messages relevant to the March. The line for the March tweets is consistently higher than that for all tweets, with the only exceptions occurring near the tail ends of the time period of interest (i.e., during the day prior to and following, the day of the March itself).

**Table 2:** Examples of sentiment score (-4 to +4) assigned to messages.

Score	Message
4	i have so many happy tears running down my face. this march is everything. women are magnificent. beautiful, smart, and strong.
	#womensmarchÂ thankful for those who stood for humanity today. thx to the men and women who stood for decency, dignity, equality & liberty!
3	an amazing and fantastic experience being part of history yesterday. #proudtobeawoman #womensmarchonwashington
	god bless you US. listen. speak out. question. fight. #liberty #justice #womensmarch
2	fewer women have unintended pregnancies, bc of places like #plannedparenthood. women having a choice is a woman's right #7in10forroe
	i can state with the utmost confidence, women do it better. #whyimarch @ the mall (washington dc)
1	i'm crying in my pajamas watching a stream of the dc #womensmarch. i wish i could be there too. we will be heard. we are stronger together.
	#womensmarch is on but it's more like a standstill since there are so many women
0	the weather didn't seem to dampen the attendance of the sf #womensmarch. #notmypresident and evan marched also. for women. for the earth. for his future. @ the mall (washington dc)
-1	woman who want abortions on the american tax payer's dime should be castrated so 'we the people' won't have to deal with their bad seeds!
	@cnnpolitics there is no single message for these women except one: the right to kill the next generation of women. #sad
-2	news flash …there is no value in declaring you are a ""nasty woman"" …it cheapens and degrads you. #disgusted #womensmarch
	go the fuck home you stupid liberals. you all have rights and nobody is taking them away from you no mater what cnn says #womensmarch
-3	woman in other countries are being tortured and can't even get an education but you US woman are mad because trump is ""mean"" wtf
	#womensmarch say you can't water bord a killer but you torture babies. these are wicked witches that torture and sell their baby body parts
-4	damn the ugly dikes at the #womensmarch need dick
	@womensmarch in dc, today i was elbowed very hard in my shoulder by a red hat man, called fat, informed i was dumb, a bitch & to die- all rh men

### Geography and sentiment

When sentiment was examined by metropolitan area (MA), mean sentiment was positive, on average, except for seven locations. (See S2 Table for the frequency of tweets per MA).

**Fig 6:**
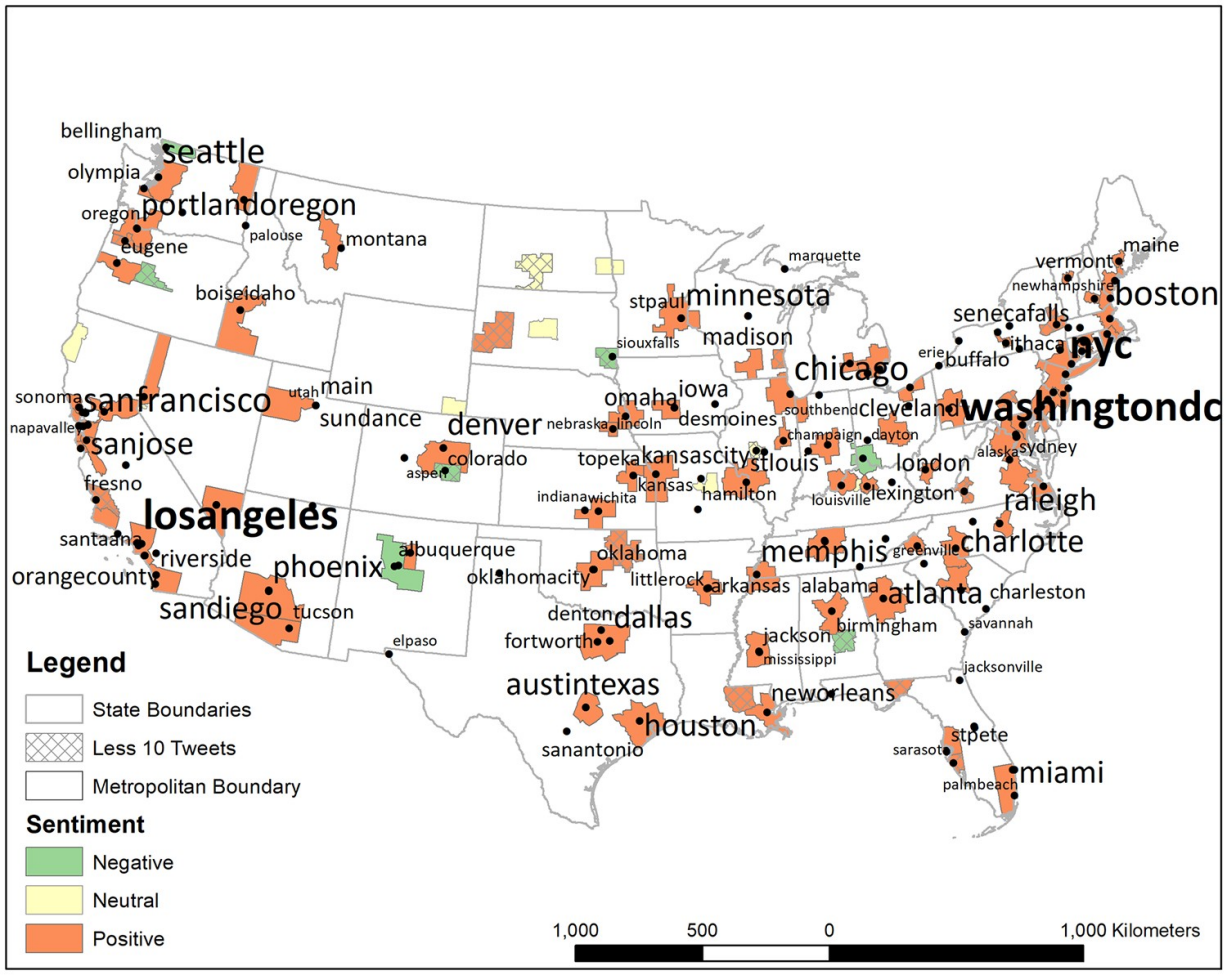
Metropolitan areas (MA) in the United States that are positive, negative and neutral (zero) in average sentiment. MA’s ranked by median. The location of the place name is based on the median location of all mentions of that place name.

As shown in Fig 6, the seven negative MAs are widely scattered, including (in alphabetical order) MAs in Alabama, Colorado, New Mexico, Ohio, Oregon, South Dakota, and Washington. The majority of these negative sentiment areas, nevertheless, tended to correspond to very small numbers of tweets (less than 10; represented by cross-hatches). For instance, although average sentiment in the MA of Bend, Oregon was negative, the mean was based on fewer than 10 tweets. There were only two MAs in which tweets relevant to the March were neutral in sentiment, on average, and these occurred in Illinois and North Dakota. Nevertheless, the sample of tweets from those neutral MAs was again quite limited in size.

**Fig 7:**
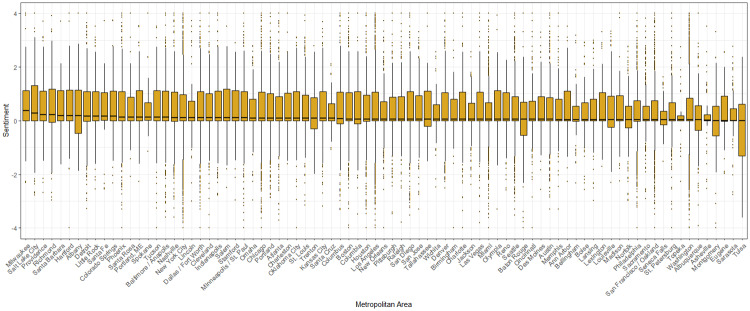
Boxplot of sentiment for each MA where more than 50 tweets were located. MA’s ranked by median sentiment score.

Next, we examined variation in sentiment for all tweets about the March for each MA with more than 50 tweets and ranked them by median sentiment (see Fig 7 for box plots of sentiment). For this subset of cities, the median for all locations tended to hover slightly above, or at, zero (Fig 7). Milwaukee had the most positive median sentiment, followed by Salt Lake City and Providence, whereas Tulsa was the least positive, with Sarasota and Eugene next in line. At locations where there is a substantial proportion of negative tweets, messages included words such as ‘*hate*, *losers*, *moron*, *resistance*’ and messages concerned topics related to prolife, religion and politics. The box plot also suggests that the distributions tend to be highly skewed, with the second quartile typically, though not always, smaller than the third quartile and with the second quartile encompassing tweets with sentiment scores close to zero. In some cases, the box plots have long whiskers. The box plots for the three largest marches, for instance, have particularly extended whiskers, demonstrating that wide-spread events are apt to generate a large sample of tweets and that these include cases at the extremes of the sentiment scores. Note, too, that certain MA’s are much smaller in size than others, which likely renders their distribution relatively small, such as that for Lincoln, Spokane, and Asheville, among others.

### Hashtags and sentiment

**Fig 8:**
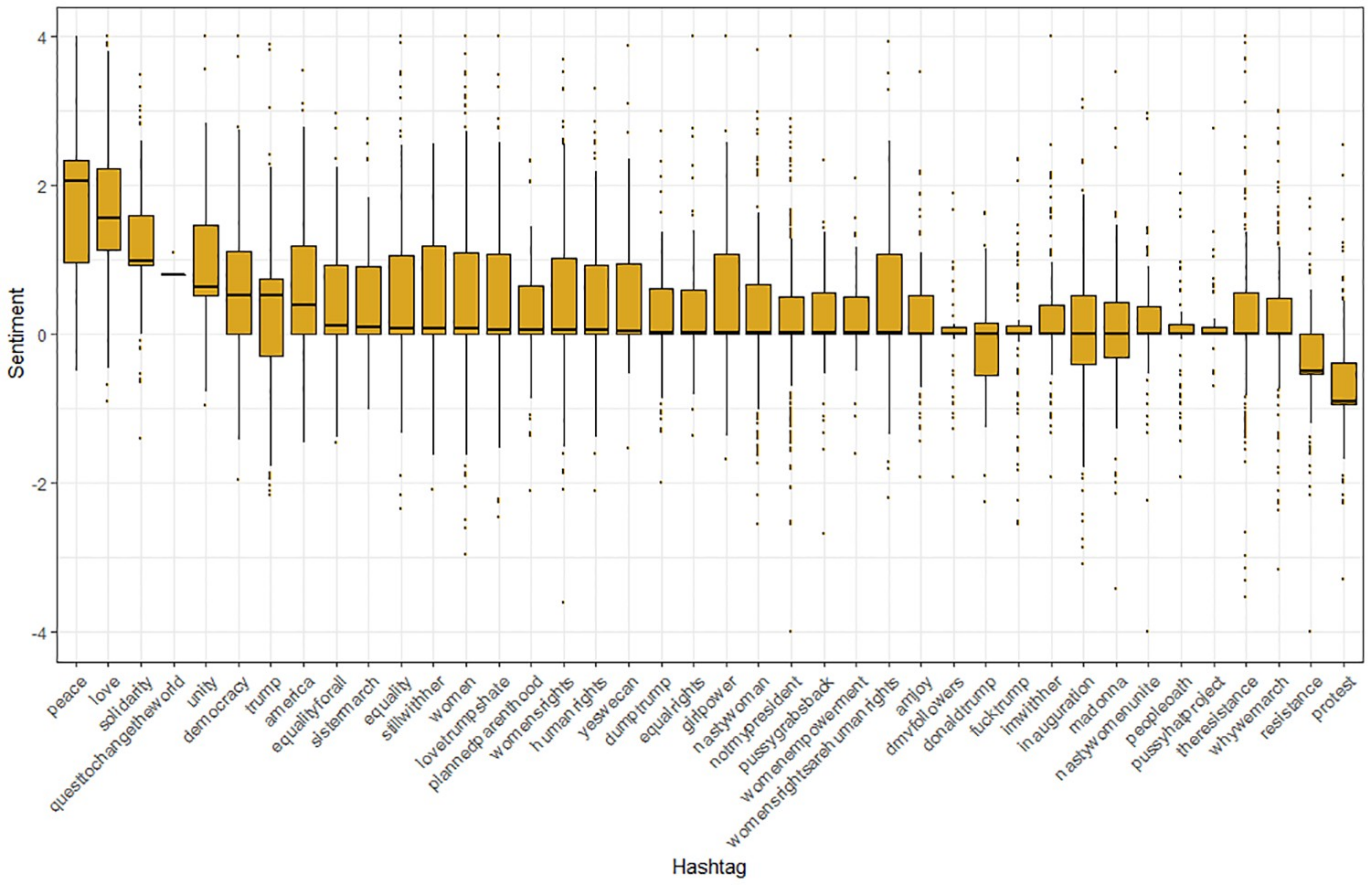
Hashtags of “why march” on the day of the Women’s March (January 21, 2019) ranked by median sentiment score (N mention > 50).

We also investigated the degree to which hashtags were relatively positive or negative in sentiment, by focusing on hashtags with over 50 mentions that were associated with “why march” on the day of the March (January 21). We ranked median sentiment for this sample of hashtags and depict the box plots for the distributions of associated sentiment in Fig 5. Messages containing hashtags such as *protest* and *resistance* were generally more negative, while those with *peace*, *love*, *solidarity*, *changetheworld*, *unity* and *democracy* were highly positive in sentiment (Fig 8). The median sentiment for the majority of hashtags hovers slightly above, or at, zero. Again, we see many skewed distributions, with whiskers that usually extend more to the positive, rather than the negative, sentiment regions.

## Discussion

The Women’s March of 2017 represented the largest single-day protest in U.S. history, with over 500,000 people attending the March on Washington, and hundreds of thousands participating in sister marches across the country and worldwide [52]. This paper examines patterns in the use, sentiment, and geographic distribution of social media during this historic event, in an examination of Twitter hashtags and tweets regarding the March. We find that online communication about the March on the social media platform of Twitter reached remarkably elevated levels on the day of the March. Tweets about the March composed a sizeable percentage of all geo-located tweets, encompassing 3.1% of tweets during the three-day period surrounding the March. By mid-afternoon on the day of the March, January 21, the percentage of tweets about the March swelled to an impressive peak of over 12% of geo-located tweets.

Not only were tweets concerning the March highly prevalent, their geographic distribution was spread widely across the continental U.S. Hashtags with place information represented over 274 names and abbreviations for various March locales, for example. Not surprisingly, tweet density appeared to correspond to the size of the local march, with the largest number of tweets emanating from, or pertaining to, the most highly populated marches, including Washington D.C., Los Angeles, and New York City. Exceptions to this density pattern suggest that metropolitan area size also influenced tweet density, with large MAs highly represented in message activity. Note that individuals did not have to attend a march in person in order to engage in electronic communication regarding the event, and therefore large metropolitan areas likely drew more social media activity than small ones.

A few states exhibited relatively low numbers of tweets about the March during this period, and they tended to be located in the low population density areas of the northern Mountain and Plain states. The population in these underrepresented states is relatively low, such as in North Dakota and Wyoming. Although there were sister marches in these states, geo-located activity on Twitter was not as prominent as in others. Note, too, that a number of people also travelled to more populous metropolitan areas, including Washington D.C., itself, and likely sent Twitter messages relevant to their destination, rather than their origin.

The overall sentiment of tweets concerning the March on January 21, 2017 was slightly positive, on average, with a mean of 0.34 and a median of 0.07 on a scale from -4 to +4. As the box-plots in Fig 7 indicate, the sentiment distribution was quite skewed in a positive direction for most MAs, with the inter-quartile range typically extending from about positive 1 to just below zero. The modal sentiment score for tweets relevant to the March, as well as tweets in general, is fairly neutral with scores around zero. We note that one of the main uses of tweets during this period was to convey basic information about the March, and therefore numerous messages were assessed as neutral in content. The event inspired negative as well as positive sentiment tweets, and therefore the overall mean of sentiment falls close to zero.

Perhaps the most notable of our findings, however, is that tweets relevant to the March were more positive in sentiment, on average, than all other geo-located tweets during the day of the March, January 21. Positive messages conveyed happiness, support, pride, and solidarity, among other affirming reactions. Moreover, upbeat tweets about the March ranged broadly across the country. The most positive hashtags included words such as peace, love, and solidarity. According to a ranking of median sentiment values, the most positive metropolitan areas were Milwaukee, Salt Lake City, and Providence. Mean tweet sentiment, rather than the median, also was positive for almost all metropolitan areas in the continental U.S.

There were exceptions to the general positive pattern, however. Seven metropolitan areas had a negative mean sentiment regarding the March (e.g., Cincinnati, Montgomery, Sioux Falls, Colorado Springs, Albuquerque, Bend, OR, and Bellingham, WA), and two areas were neutral (e.g., Bismarck, ND and Springfield, IL). Nonetheless, the majority of the negative and neutral locations emanated from areas with fewer than 10 tweets (6 of 9). Thus, the values for the measures of central tendency should not be considered a reliable gauge of sentiment in these metropolitan areas.

Finally, our analysis uncovered relatively little evidence of systematic, aggressive, bullying tweets that targeted the March or its participants. Moreover, as just discussed, no extensive geographical areas within the continental U.S. revealed consistently negative messages. Negative tweets did occur, of course, with some of the least positive themes in hashtags involving “protest” and “resistance.” Messages with the most extremely, negative sentiment scores included those with insulting attacks aimed at the protest, and there were cases of hashtags and tweets that contained vicious attacks on the marchers. Yet some negative tweets also appeared to emanate from the March supporters, themselves, who were angrily airing their political and social complaints.

This pattern of relatively low levels of negative Twitter communication about the March remains surprising, given the ubiquitous, and extensive levels of bullying documented in social media [16] and the high rates of aggressive messages aimed at women [21]. Therefore, the dampened level of negative sentiment regarding the Women’s March remains particularly notable and represents further proof of the overall, supportive and buoyant nature of the protest.

Our study has clear strengths, but it is not without limitations. First, sentiment analysis remains part art, with substantial room for misinterpretation. Our sentiment classifier has the advantage that it was designed for use with this data set, and its reliability scores are relatively high. Yet tweet sentiment does not necessarily reflect actual attitudes, and sarcasm, mixed messages, and other frequent, tricky communication patterns also leave open room for error in classification. Second, our information about the locations of, and number of attendees at, various marches was obtained from Wikipedia, and subject to misreporting. Our analysis also was limited to tweets that included identifiable, geographic information. If people with the most deleterious messages attempted to hide their location, then the sample of tweets used here could be more positive, overall, than one that consisted of all messages about the March, regardless of geographical information. In addition, we know relatively little about the motivations behind positive and negative reactions to the March, and future work would benefit from a detailed content analysis of the messages. Finally, generalizations drawn from our study are limited formally to the population of Twitter users, who tend to be younger, more urban, and more diverse racially than the general U.S. population [53].

In conclusion, the Women’s March of 2017 generated a considerable number of upbeat Twitter messages over a short period of time. The distribution of these tweets was impressive, with extensive MA’s overrepresented, but with activity in hundreds of small geographic locations as well. We detected only limited evidence of a backlash of negative content, although exceptions arose, particularly in underrepresented, geographic locations. We see here that the Women’s Movement that began decades earlier did not disappear, but along with the joint resources of other organizations, re-emerged in what appears to have been a resoundingly successful March on Washington in 2017.

## Supporting information

S1 Table List of hashtags (a) associated with the march and (b) describing the march (frequency > 15). (DOCX)

S2 Table Number of tweets per MA. (DOCX)
