# Supplementary material for: The geography of sentiment towards the Women’s March of 2017
Source: PLoS One. 2020 Jun 4;15(6):e0233994. doi: 10.1371/journal.pone.0233994 (PMC7272063; doi:10.1371/journal.pone.0233994)
Supplement: S1 Table — (DOCX) [file pone.0233994.s001.docx]

**S1 Table. List of hashtags (a) associated with the March and (b) describing the March (frequency > 15).**

| **a) Hashtags: associated with the march (womensmarch)** |
| --- |
| womensmarch,womensmarchonwashington,womensmarchla,womansmarch,womensmarchnyc,womensmarch2017,marchonwashington,womensmarchlosangeles,womensmarchchicago,womensmarchchi,boswomensmarch,bostonwomensmarch,atlantamarch,womensmarchsf,womensmarchdenver,womenmarch,nycwomensmarch,womensmarchaustin,womensmarchonnyc,womensmarchseattle,womensmarchmn,womxnsmarchseattle,womensmarchoakland,womensmarchboston,womansmarchonwashington,womensmarchpdx,womensmarchdc,sistermarch,marchonaustin,millionwomenmarch,womensmarchlv,womensmarchsandiego,seattlewomxnsmarch,womensmarchphiladelphia,womensmarchatlanta,womensmarchsacramento,houstonwomensmarch,womansmarch2017,seattlewomensmarch,mensmarch,dallaswomensmarch,womensmarchatx,womensmarchonchicago,millionwomanmarch,womensmarchmadison,marchonmain,womenmarchonwashington,womensmarchmiami,womensmarchonaustin,sfwomensmarch,womensmarchphilly,marchonlansing,womensmarchondenver,oaklandwomensmarch,phillywomenmarch,womensmarchraleigh,womensmarchwashington,womensmarchsanjose,womensmarchkc,memphiswomensmarch,womensmarchsanfrancisco,womxnsmarch,womansmarchla,womensmarchlondon,womensmarchphoenix,chicagowomensmarch,denverwomensmarch,lawomensmarch,sandiegowomensmarch,womensmarchindy,womensmarchonphiladelphia,womensmarchportland,womensmarchhouston,womensmarchsd,womensmarchclt,womensmarchstl,womensmarchok,womensmarchonwashington,sdwomensmarch,womensmarchdallas,womensmarchmia,womensmarchbayarea,ocwomensmarch,womensmarchomaha,womensmarchiowa,womensmarchjxn,womensmarchnola,womensmarchpeoria,womensmarchstpete,marchforwomen,womensmarchminnesota,womensmarchnashville,womensmarchoc,womensmarchoma,womensmarchorlando,cltmarch,womensmarchhartford,womensmarchonraleigh,womensmarchonboston,womensmarchonriversideca,womensmarchsigns,womensmarchny,womensmarchonlosangeles,womensmarchriverside,womanmarch,womensmarchindianapolis,womensmarchmemphis,womensmarchnc,womensmarchsac,womensmarchsj |
| **b) Hashtags: reasons for the march (whymarch)** |
| whyimarch,notmypresident,lovetrumpshate,womensrights,resist,women,equality,nastywoman,womenwhohaveinspiredme,theresistance,inauguration,womensrightsarehumanrights,nastywomen,trump,strongertogether,whywemarch,humanrights,love,protest,solidarity,riseup,girlpower,imwithher,feminism,dumptrump,resisttrump,pussygrabsback,pussypower,fucktrump,equalityforall,blacklivesmatter,wethepeople,thefutureisfemale,thisiswhatdemocracylookslike,feminist,democracy,amjoy,equalrights,nastywomenunite,job,resistfromday1,peace,imarchfor,stillwithher,madonna,unity,peopleoath,womenempowerment,womenoftheworld,womenmobilizenc,plannedparenthood,noisymajority,yeswecan,istandwithpp,questtochangetheworld,pussyhat,donaldtrump,dmvfollowers,prolife,unitedwestand,imstillwithher,cc17,hope,woman,standup,proud,civilrights,cnn,freedom,lovearmy,respect,powertogethertn,repost,womeninstem,nastywomanvote,notmypotus,mybodymychoice,lovewins,marchforlife,hiring,united,womenintech,potus,truth,womenpower,trumpinauguration,2017,nasty,lgbt,pussyhats,strongwomen,hearourvoice,strongertogetherkc,addhername,ashleyjudd,womenrights,trumpprotest,trumppresident,loveislove,wewontgoback,presidenttrump,pink,rights,revolution,climatechange,heretostay,peacefulprotest,signs,herstory,nevertrump,protestsigns,womensrallyindy,powertothepeople,nastywomenmakehistory,lgbtq,diversity,bethechange,hillaryclinton,westandwithyou,msnbc,marchon,history,antitrump,beanengineer,impeachtrump,decision2016,unitedagainsthate,power,rally,firedup,ncvrwindc,wonderwoman,lovenothate,sundance2017,broadwaystrong,fightlikeagirl,inauguration2017,equalrightsforall,unite,justice,fakenews,nofilter,vivalavulva,wemarchwithyou,notonestepback,biggestlittlemarch,reproductiverights,werise,sisters,starwars,empowerment,freemelania,americafirst,princessleia,togetherwerise,wemarch,staywoke,notmymarch,seanspicer,sisterhood,standwithpp,whyimarchnyc,socialjustice,firedupreadytogo,pussy,obama,democrats,sisepuede,indivisible,election2016,westandunited,thispussygrabsback,life,news,pussup,equalpay,inspired,whitehouse,womansrights |
