# Supplementary material for: The geography of sentiment towards the Women’s March of 2017
Source: PLoS One. 2020 Jun 4;15(6):e0233994. doi: 10.1371/journal.pone.0233994 (PMC7272063; doi:10.1371/journal.pone.0233994)
Supplement: S2 Table — (DOCX) [file pone.0233994.s002.docx]

**S2 Table. Number of tweets per MA.**

| **MA’s** | **Number of Tweets** |
| --- | --- |
| Washington | 9438 |
| New York City | 5973 |
| Los Angeles | 5699 |
| San Francisco / Oakland | 2495 |
| Chicago | 2368 |
| Boston | 1689 |
| Seattle | 1505 |
| Philadelphia | 1400 |
| Austin | 1190 |
| Dallas / Fort Worth | 1037 |
| Denver | 928 |
| Atlanta | 870 |
| Miami | 858 |
| San Diego | 855 |
| Portland | 835 |
| Houston | 823 |
| Phoenix | 698 |
| Minneapolis / St. Paul | 678 |
| Baltimore / Anapolis | 592 |
| Nashville | 553 |
| San Jose | 482 |
| Indianapolis | 472 |
| Sacramento | 469 |
| Detroit | 447 |
| Las Vegas | 446 |
| Columbus | 445 |
| St. Louis | 387 |
| St. Petersburg | 379 |
| Charlotte | 375 |
| Kansas City | 348 |
| Raleigh | 334 |
| New Orleans | 318 |
| Cleveland | 304 |
| Pittsburgh | 298 |
| Cincinnati | 290 |
| Oklahoma City | 252 |
| Providence | 192 |
| Columbia | 191 |
| Albuquerque | 182 |
| Hartford | 181 |
| Norfolk | 175 |
| Des Moines | 173 |
| Little Rock | 171 |
| Omaha | 169 |
| Memphis | 164 |
| Lansing | 154 |
| Stamford | 141 |
| Ann Arbor | 137 |
| Tucson | 137 |
| Richmond | 135 |
| Louisville | 133 |
| Jackson | 125 |
| Baton Rouge | 115 |
| Salt Lake City | 115 |
| Sarasota | 112 |
| Boise | 111 |
| Tallahassee | 107 |
| Trenton | 103 |
| Birmingham | 98 |
| Eugene | 98 |
| Madison | 96 |
| Portland, ME | 96 |
| Reno | 93 |
| Milwaukee | 92 |
| Montgomery | 91 |
| Colorado Springs | 88 |
| Santa Rosa | 79 |
| Asheville | 78 |
| Lincoln | 78 |
| Olympia | 78 |
| Santa Cruz | 69 |
| Seneca Falls | 67 |
| Santa Barbara | 66 |
| Santa Fe | 66 |
| Bellingham | 64 |
| Albany | 63 |
| Salem | 62 |
| Tulsa | 61 |
| Topeka | 60 |
| Lexington | 58 |
| Wichita | 57 |
| Charleston | 50 |
| Spokane | 50 |
